# Atypical Neuroimaging Manifestations of Linear Scleroderma “en coup de sabre”

**Published:** 2015

**Authors:** Andrew M. ALLMENDINGER, Joseph A. RICCI, Naman S. DESAI, Narayan VISWANADHAN, Diana RODRIGUEZ

**Affiliations:** 1 The Department of Radiology, Brigham and Women’s Hospital, Harvard Medical School, Boston, MA, USA; 2 The Division of Plastic Surgery, Brigham and Women’s Hospital, Harvard Medical School, Boston, MA, USA; 3 The Division of Neuroradiology, Boston Children’s Hospital, Harvard Medical School, Boston, MA, USA

**Keywords:** Linear Scleroderma “en coup de sabre”, Localized scleroderma, Morphea, Cerebellum, MRI

## Abstract

Linear scleroderma “en coup de sabre” is a subset of localized scleroderma with band-like sclerotic lesions typically involving the fronto-parietal regions of the scalp. Patients often present with neurologic symptoms. On imaging, patients may have lesions in the cerebrum ipsilateral to the scalp abnormality. Infratentorial lesions and other lesions not closely associated with the overlying scalp abnormality, such as those found in the cerebellum, have been reported, but are extremely uncommon. We present a case of an 8-year-old boy with a left fronto-parietal “en coup de sabre” scalp lesion and describe the neuroimaging findings of a progressively enlarging left cerebellar lesion discovered incidentally on routine magnetic resonance imaging. Interestingly, the patient had no neurologic symptoms given the size of the mass identified.

## Introduction

Linear scleroderma “en coup de sabre” (ECDS) is a rare subset of localized scleroderma. Affected individuals typically have a characteristic atrophic skin lesion involving the fronto-parietal scalp. The disease usually has a benign course and has been distinguished in the past from systemic scleroderma by lack of significant internal organ involvement (1). However, on rare occasion evidence of organ involvement in linear scleroderma can be manifested as involvement of the rheumatologic, neurologic, and ophthalmologic systems (2). Additionally, rare neurologic symptoms can be seen associated with linear scleroderma. The most common neurologic symptom is epilepsy, but other neurologic deficits like movement disorders or behavioral changes have been reported (2). The presence of neurologic symptoms often heralds the existence of an intracranial abnormality; it is unusual for these patients to have a mass without the presence of neurological symptoms. Intracranial magnetic resonance imaging (MRI) findings seen in this group of patients commonly include: focal brain atrophy, calcifications and T2-hyperintense white matter lesions that may demonstrate contrast enhancement (2,3). Characteristically, white matter lesions and calcifications are found in the cerebral hemisphere ipsilateral to the skin abnormality. Although rare, infratentorial lesions and lesions located away from the skin manifestation have been reported in the literature (2,4,5). This report describes the case of a young boy with linear scleroderma ECDS, who was found to have a progressively enlarging cerebellar lesion on incidental MRI, in the absence of any neurological signs and symptoms.

## Case Report

**Fig 1:**
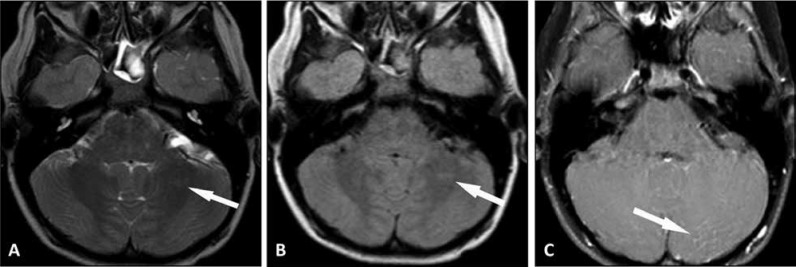
Initial brain MRI exam. (A) Axial T2-weighted image, (B) axial FLAIR image showing subtle 10 x 4 mm focus of T2 prolongation in the white matter of the left cerebellar hemisphere and (C) mild diffuse leptomeningeal enhancement was present overlying the left posterior cerebellar hemisphere.

**Fig 2:**
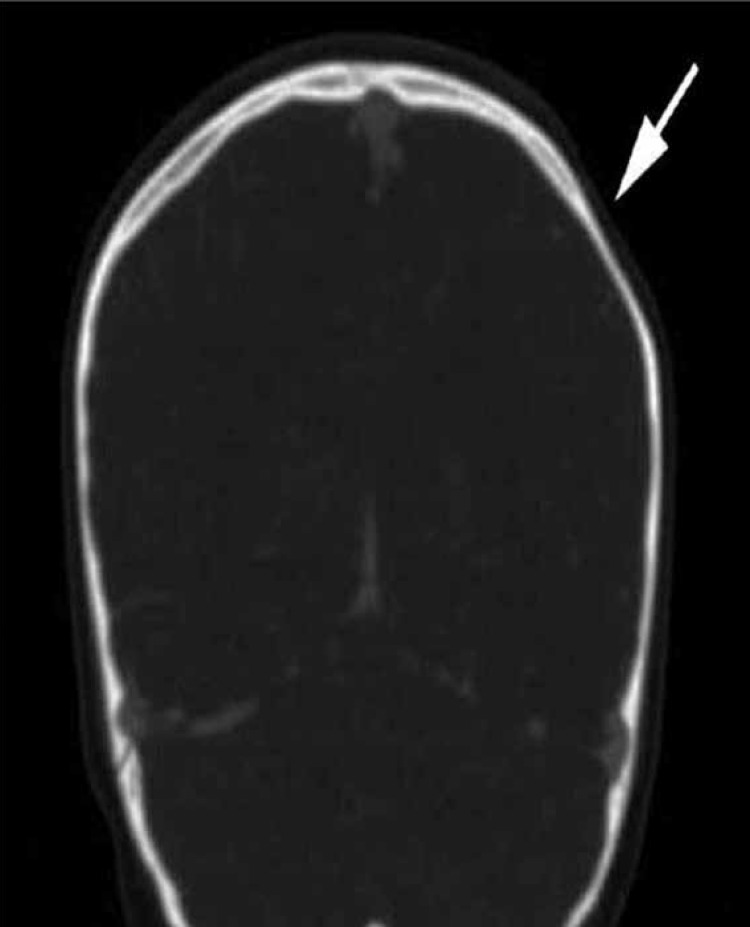
Coronal 2-D reformatted CT image with contrast demonstrates focal scalp thinning within the left fronto-parietal region and thinning of the underlying frontal and parietal bones.

**Fig 3:**
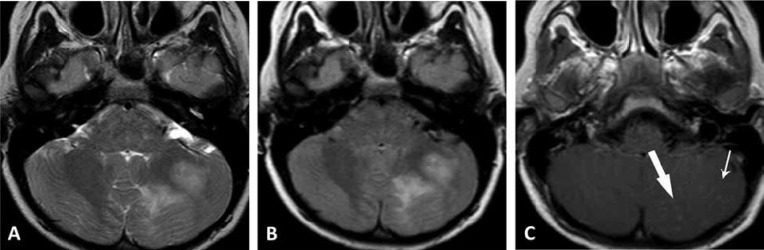
Repeat MRI fourteen months later. (A) Axial T2-weighted image, (B) axial FLAIR image showing new, extensive, patchy areas of T2 prolongation in the white matter of the left cerebellar hemisphere and (C) post contrast axial T1-weighted image demonstrating persistent mild diffuse leptomeningeal enhancement (small arrow) with new areas of nodular parenchymal enhancement (large arrow) in the left cerebellar hemisphere.

**Fig 4:**
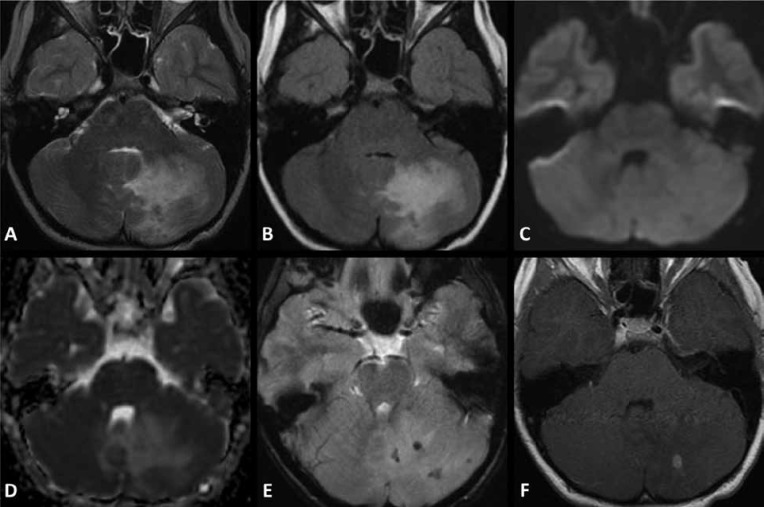
Third MRI at age eight. (A) Axial T2- weighted image, (B) axial FLAIR image showing continued increase in size of the patchy areas of T2 prolongation in the left cerebellar hemisphere, (C) axial diffusion weighted image, (D) axial ADC map show corresponding increased diffusivity of the parenchymal lesions, (E) small foci of increased magnetic susceptibility on GRE T2* images are present and (F) post contrast axial T1-weighted image showing persistent leptomeningeal enhancement with more confluent nodular parenchymal enhancement.

A 5-year-old boy was referred for routine brain MRI by the department of neurosurgery after presenting for consultation in the setting of his primary illness, linear scleroderma ECDS, affecting his left fronto-parietal scalp. The patient was initially diagnosed with linear scleroderma at that time on the basis of physical exam findings. The diagnosis was confirmed via a biopsy of his left fronto-parietal scalp. He subsequently received treatment with methotrexate weekly for 22 months. Prior to his diagnosis of linear scleroderma ECDS, the child had no other relevant past medical history. At the time of diagnosis, as well as at the time of the first MRI, the patient had no neurologic deficits or neurologic symptoms. On physical examination, the patient had a band-like scalp lesion in the left upper fronto-parietal region consisting of a 7.5 x 2.5 cm atrophic, shiny, pink-white plaque with alopecia and hyperkeratosis. The patient was noted to have a smaller lesion of similar characteristics, anterior and inferior to the large plaque. Both the patient and his mother reported that these lesions had improved with initial treatment and had appeared stable in size since the discontinuation of the medication. Brain MRI was performed using a 1.5- T MR scanner (Signa HDx; GE Healthcare, Waukesha, WI, USA). At the time of diagnosis, imaging demonstrated a single, ill defined, non-enhancing, 10 x 4 mm focus of T2 prolongation in the left cerebellar white matter (Figures 1A & 1B). On post contrast images, there was mild diffuse leptomeningeal enhancement in the posterior aspect of the left cerebellar hemisphere (Figure 1C). Subsequently, a head CT scan demonstrated a focal area of scalp thinning within the left fronto-parietal region with associated flattening and thinning of the underlying frontal and parietal bones. There was no evidence of intracranial calcifications (Figure 2). A follow-up brain MRI was performed 14 months later, at age six, while the patient was on treatment with methotrexate, demonstrated new patchy, large, confluent areas of T2 prolongation in the white matter of the left cerebellar hemisphere without significant mass effect. On diffusion weighted imaging (DWI) these parenchymal abnormalities showed increased diffusivity. Post contrast images demonstrated new scattered nodular foci of enhancement throughout these areas of parenchymal signal abnormality with corresponding susceptibility effect, most compatible with micro-hemorrhages (Figures 3A & 3B). The degree of leptomeningeal enhancement in the left cerebellar hemisphere had increased since the previous study (Figure 3C). The third and most recent brain MRI was performed at eight years of age (twelve months after the prior exam) and demonstrated continued increase in size of the confluent areas of T2 prolongation in the left cerebellar hemisphere (Figures 4A & 4B). Treatment with methotrexate had been discontinued three months prior to obtaining this most recent brain MRI. These lesions again demonstrated increased diffusivity on DWI (Figures 4C & 4D). Additionally, new areas of T2 prolongation were identified involving the cerebellar vermis and the medial aspect of the right cerebellar hemisphere. Post contrast images showed more confluent nodular parenchymal enhancement and persistent diffuse leptomeningeal enhancement (Figure 4E). Corresponding magnetic susceptibility on gradient-recalled echo (GRE) T2* was present within the nodular foci of enhancement (Figure 4F).

## Discussion

Linear scleroderma “en coup de sabre” is a rare subset of localized scleroderma, a distinct and separate disease entity from systemic scleroderma (1). Localized scleroderma is also referred to as Morphea, and is differentiated from systemic sclerosis by the absence of sclerodactyly, Raynaud’s phenomenon, capillaroscopic abnormalities, and organ involvement. Localized scleroderma is a fibrosing condition characterized by thickening and hardening of the skin as a result of increased collagen production, with involvement of the subcutaneous tissue and underlying bone. Five clinical subtypes of localized scleroderma have been suggested: circumscribed, linear, generalized, pansclerotic, and mixed (1). Linear scleroderma is the most common subtype in children and has three clinical variants: linear limb involvement, “en coup de sabre” (ECDS), and progressive hemifacial atrophy (Parry- Romberg syndrome) (1,6). Underlying tissue atrophy is commonly present in these three variants. ECDS is defined by a unilateral band of sclerotic skin lesions in the fronto-parietal area of the head resembling a deep saber wound, hence the name “en coup de sabre” (meaning, the saber cut in French). ECDS can be associated with ipsilateral underlying intracranial lesions and ocular involvement (1). Progressive hemifacial atrophy (PFH) consists of atrophy which extends below the skin and subcutaneous tissues, and often involves underlying muscle and bone (2). Several authors have postulated that ECDS and progressive hemifacial atrophy (PHA) may be clinical variants of the same disease (1-3,6). These two entities can coexist and share similarities including a comparable age of onset, female predominance, identical neurologic and ophthalmologic complications, and the same neuroimaging characteristics. Both diseases may respond to immunosuppressive treatment (2). The incidence of localized scleroderma has been reported as 0.4 to 2.7 per 100,000 people (1), with a female predominance (6), and increased prevalence in the white population (1). The prevalence of localized scleroderma seems to be equal in adults and children. A large, multicenter, multinational study reported a mean age at disease onset of 7.3 years (range 0 – 16 years). The mean time between the first manifestation of the disease and diagnosis was 1.6 years, with a median of eleven months (range 0 – 16.7 years) (6). The skin lesions of localized scleroderma (Morphea) are a characteristic clinical finding which undergo an initial inflammatory stage of erythematous, dusky, violaceous patches or plaques. Later, the center of the lesion becomes white and sclerotic, with a surrounding outer “violaceous ring”. Once the active phase resolves, the skin lesion turns into a near completely white sclerotic plaque with subsequent post-inflammatory hyperpigmentation. Excessive collagen deposition destroys hair follicles and adnexal structures, resulting in hairless, anhidrotic plaques (1). Aside from alopecia in a band-like fashion in the fronto-parietal scalp and forehead, cutaneous manifestations may extend to the nose, cheek, chin, and neck. Facial atrophy occurs if the underlying muscle, cartilage and bone are involved. These clinical findings can overlap or coexist in ECDS and PFH making it difficult to classify them as separate entities (2,6). In most reported cases of linear scleroderma in the literature, neurologic symptoms are usually preceded by the skin manifestations; however, neurologic symptoms can sometimes appear first. The range of neurologic symptoms associated with craniofacial scleroderma is variable. They include seizures, recent onset headaches, focal neurologic deficits and movement disorders which can be secondary to brain lesions, trigeminal neuralgia, masticatory spasms, mimics of hemiplegic migraines, behavioral changes, or progressive intellectual deterioration due to cerebral hemiatrophy either with or without focal seizures. Rarely, even death has been reported due to brainstem involvement (2,6). Epilepsy remains, however, the most common neurologic symptom associated with linear scleroderma (2). Typically, patients with linear scleroderma intracranial pathology, seen on neuroimaging studies will present with neurological symptoms. Uncommonly, asymptomatic patients may be found to have abnormal neuroimaging studies. Classic central nervous system findings on CT and MRI include brain parenchyma atrophy, white matter lesions, and focal subcortical calcifications. Parenchyma and leptomeningeal enhancement may also be seen (2,3). Neuroimaging findings are typically ispilateral to the skin lesions in the cerebral hemisphere. Rarely, contralateral and infratentorial involvement have been described (2,4,5). Calcifications typically involve the basal ganglia, thalami, and dentate nuclei, but can also be found in the subcortical white matter (2,7). MRI usually exhibits T2 hyperintensities, mostly in the subcortical white matter, but also in the corpus callosum, deep grey nuclei, and brain stem. Cerebral atrophy is generally focal and subtle, characterized by blurring of the gray-white interface, cortical thickening, and abnormal gyral pattern (2,3). Rarely, hippocampal atrophy has been reported (7). The diagnosis of linear scleroderma is made based on the clinical characteristics of the cutaneous and soft tissue findings. There are no laboratory tests diagnostic for linear scleroderma, although, patients may be positive for anti-nuclear antibodies (ANA), anti-single-strandedDNA antibodies (anti-ssDNA) and rheumatoid factor (2,6). Histopathologic examination cannot differentiate localized scleroderma from systemic sclerosis. In the early stage both entities have lymphocytic perivascular infiltration in the reticular dermis and swollen endothelial cells. Late stages of disease show thickened collagen bundles infiltrating the dermis and extending into the subcutaneous fat (1). The etiology of linear scleroderma is not well understood; however, there is evidence suggesting an autoimmune origin of this disease (2). Early in the disease course, damage to the endothelial cells results in increased vascular permeability with mononuclear cell infiltration, perivascular inflammatory cell infiltrates, vascular intimal thickening, and vessel narrowing. These vessels gradually lose their elasticity, and the media and adventitia become fibrotic (8). Pathogenesis of CNS disease suggests perivascular infiltrate and vasculitis, but is limited, as biopsies are not routinely done. In a few reported cases, histological findings include gliosis, leptomeningeal band-like sclerosis, thickened blood vessel walls, and intraparenchymal calcifications, all of which suggests a chronic inflammatory process (8). Environmental factors have been reported occurring close to the disease onset, including mechanical factors, infections, drugs and psychological distress (6). This case is unique in that the patient‘s CNS manifestations were not located in the cerebrum underlying the skin lesion, but were infratentorial in the contralateral cerebellar hemisphere. More interestingly however, the patient in this case did not manifest with any neurologic symptoms despite the fact that he had progression of his intracranial pathology over the span of several years. In rare cases of cerebellar involvement, the disease usually manifests as atrophy and volume loss. On the contrary, our patient presented with infiltrative appearing lesions that exerted mild mass effect on the fourth ventricle. Furthermore, despite immunosuppressive treatment, these white matter cerebellar lesions increased in size considerably during a 2-year period. In the absence of the patient‘s history one could easily confuse the findings with infiltrating tumor. However, it would be unlikely for a primary CNS neoplasm to present initially with mainly leptomeningeal enhancement. Several of the lesions demonstrated nodular enhancement on post contrast T1-weighted images with corresponding magnetic susceptibility, suggesting injured vascular wall with presence of micro-hemorrhages. The parenchymal lesions demonstrated increased diffusivity most likely consistent with insterstitial edema. The constellation of neuroimaging findings seen in this case are suggestive of cerebral vasculopathy, classically seen as part of other autoimmune processes. Despite progression of neuroimaging findings and given that the patient had a normal clinical exam with absence of neurologic symptoms, a conservative approach was preferred and biopsy of the cerebellar lesion was not performed. A period of observation was suggested to the patient, whose family was agreeable, and he has not had any neurological manifestations to date.

**In conclusion**, typical neuroimaging features of linear scleroderma including atrophy, white matter lesions and calcifications typically involve the cerebral hemisphere ipsilateral to the skin lesion. However, CNS lesions can also present away from the skin lesion in the cerebellar hemisphere with imaging features similar to a demyelinating process or infiltrating tumors, as opposed to the more common presentation of parenchymal atrophy. It is important to recognize that even in the absence of neurological symptoms, patients with linear scleroderma may present with atypical neuroimaging features found on routine imaging which should not be mistaken for an infiltrating neoplasm.

## Notes

**Authors’ Contributions:** All authors have contributed equally to the care of this patient and to the preparation of this manuscript. All authors agree with its content.
